# Another choice for measuring tree photosynthesis *in vitro*

**DOI:** 10.7717/peerj.5933

**Published:** 2019-01-08

**Authors:** Changjun Meng, Xiao Liu, Yongfu Chai, Jinshi Xu, Ming Yue

**Affiliations:** 1 Key Laboratory of Resource Biology and Biotechnology in Western China, Northwest University, Xi’an, Shaanxi, China; 2 College of Biology and Environmental Engineering, Xi’an University, Xi’an, Shaanxi, China; 3 School of Life Sciences, Northwest University, Xi’an, Shaanxi, China

**Keywords:** Beveling method, Cracking method, Cut branches, Gas exchange parameters, Tree species

## Abstract

**Background:**

In the case of tall trees in the field or in rugged terrain where an instrument cannot be placed operationally, beveling is a popular method used to measure *in vitro* photosynthesis. However, some studies and our own research have shown that net photosynthesis values measured *in vitro* are generally significantly lower than values measured *in situ*.

**Methods:**

To develop a more accurate and applicable method for *in vitro* determination of photosynthesis, we evaluated five different methods for preparing detached tree branches to measure photosynthesis and gas exchange *in vitro* (beveling, cracking, splitting, girdling, and immersion in salicylic acid solution). Ten common tree-species were used.

**Results:**

By comparing light response curves and water-status data, we found that (1) it is possible, to some extent, to substitute *in vitro* measurement of photosynthetic characteristics of tree species for *in situ* measurement, provided a suitable treatment is employed; (2) the beveling method is likely to underestimate photosynthetic potential of some trees; (3) after cracking application, most detached branches effectively continued to absorb water; and (4) measurements obtained using detached tree-branches processed by the cracking method were closer to those obtained *in situ* in intact trees; (5) some tree species (*Diospyros kaki, Eriobotrya japonica*) appeared to be particularly sensitive to the cracking method, and their *in-vitro* maximum net photosynthesis rate (*P*_max_) was significantly less than the *in-situ* value (*P* < 0.05).

**Discussion:**

Our findings provide a methodological support for comprehensive and accurate measurement of plant functional traits. The use of the cracking method contributes to feasibility and reliability of the measurement of photosynthetic parameters in tall trees, thus providing more accurate photosynthetic parameters for the analysis of trade-off strategies at the leaf level.

## Introduction

Photosynthesis is the most basic activity in plants (Ashraf & Harris, 2013) and is crucial for many processes such as plant growth, biomass allocation, species competition as well as ecosystem function (Ruimy & Bondeau, 1999; Dai et al., 2017). Owing to their fundamental role, photosynthetic parameters are regarded as indispensable components of plant functional traits. Plant functional traits can be divided into soft traits (growth type and life form) and hard traits (relative growth rate and photosynthetic capacity) on the basis of the difficulty observed in rapid measurement and quantitative description (Cornelissen et al., 2003). Gas exchange parameters are some of the hard traits among plant functional variables, especially *P*_max_ (maximum net photosynthesis rate), which reflects photosynthesis potential of a species and is the core trait of the leaf economy spectrum (Wright et al., 2004). However, in the case of trees, *in situ* measurement carried out using branches and leaves still attached to the tree is often difficult. Consequently, some studies focused only on photosynthesis of saplings or seedlings (Gallé, Haldimann & Feller, 2007; Mayoral et al., 2015; Harmens et al., 2017), whereas many studies tend to choose soft traits, which are easier to measure (e.g., Hérault et al., 2011). Although some soft traits are closely related to some of the hard traits, the extent of equivalence among them does not allow for uncontroversial, consistent results. Therefore, *P*_max_ of adult trees needs to be measured more reliably for irrefutable consideration among plant functional traits.

Branch beveling is currently a popular method for measuring *P*_max_ of tall trees *in vitro*, where the leaf’s petiole remains attached to the branch but the branch is cut from the tree (Chai et al., 2015). Koike & Sakagami (1984) and Koike (1986) found that beveling treatment could significantly increase the water-absorbing area of branches and that the method was suitable to determine *in vitro* photosynthesis in some plants. Tang & Wang (2011) aimed to develop a better approach to measure *in vitro* photosynthesis in adult trees. They compared beveling of branches with phloem girdling, and found it was more feasible to girdle the phloem about 3 cm from the cut and remove most leaves. Increase in the water absorption area of detached branches is an important issue to be considered in the measurement of in-vitro photosynthesis. Although the splitting method (branch end was split) and cracking method (branch end was cracked) are rarely used for *in-vitro* photosynthesis, they are still beneficial to increase the water absorption area of detached branches. In particular, the accuracy of measurements of photosynthetic parameters may sometimes be significantly affected in cases where the water supply to its leaves is not timely and effective after the tree branch has been cut off (Luo, Zhang & Zhang, 2016).

In fact, some studies (e.g., Gauthier & Jacobs, 2018) have shown that net photosynthesis (A) values measured *in vitro* after beveling, decreased by about 40–70% compared to those obtained from *in situ* measurements, likely owing to a water deficit. Studies have also shown that there were significant differences in the effects of the beveling method and other *in-vitro* methods on the water supply capacity of *in-vitro* branches. Compared with the traditional beveling method, the transpiration rate(Tr) of the detached branches treated with the girdling method was significantly higher than that of the oblique cutting method, and the Tr of the girdling method was about 15% higher than that of the beveling method (Tang & Wang, 2011). Water deficit can affect photosynthesis in different ways (Chaves, 1991; Matta, Maestri & Barros, 1998; Flexas et al., 2004; Pinheiro & Chaves, 2011). First, water deficit often makes the xylem conduit of the excised twigs produce cavitation and embolism (Gullo & Salleo, 1992), which leads to a decline in the water transport capacity of the xylem. Moreover, it is possible for the photosynthetic machinery to be damaged through either stomatal or non-stomatal limitations as water supply becomes reduced (Wilson, Baldocchi & Hanson, 2000; Flexas & Medrano, 2002; Huseynova et al., 2016), causing partial or complete stomatal closure, which in turn could result in a decrease in transpiration rate (Saliendra, Sperry & Comstock, 1995; Boyle, Mcainsh & Dodd, 2015) and, concomitantly, in a decrease of CO_2_ uptake (Nagy et al., 1998; Grantz et al., 2016), intracellular CO_2_ concentration, and CO_2_ assimilation rate (Gimenez, Mitchell & Lawlor, 1992; Lawlor & Cornic, 2002). The decline in net photosynthetic CO_2_ uptake caused by water deficit could reduce availability of soluble sugars, thereby limiting leaf dark respiration (Rd) (Rodríguez-Calcerrada, Shahin & Rambal, 2011). Photosystem II (PSII) is also vulnerable to water deficit. When PSII activity is limited, the balance between generation and utilization of electrons will be disturbed and quantum yield will change significantly (Matta, Maestri & Barros, 1998; Vanlerberghe, Martyn & Dahal, 2016).

Obviously, an effective and uninterrupted water supply is an important aspect for selecting the optimal method for accurate *in vitro* determination of *P*_max_ and dark respiration. Some physical and chemical methods have been applied aiming to preserve woody flowers via an increase in water absorption area of* in vitro* branches, whereas at the same time preventing mucilaginous secretions by blocking xylem vessels, controlling transpiration, and maintaining leaf vigor (Conrado, Shanahan & Eisinger, 1980; Rai, Sharma & Sharma, 1986; Bar-Yosef & Lieth, 2013). For example, the cracking method (ends of branches are cracked) and the splitting method (ends of branches are split, then a small stone is inserted into the incision) could increase the absorption area of detached branches effectively. On the other hand, the salicylic acid method (trimmings are immersed in salicylic acid solution) can influence stomatal opening and also inhibit bacteria (Manthe, Schulz & Schnabl, 1992). Nevertheless, these treatments are rarely used in preparation for *in vitro* measurement of photosynthesis. Therefore, we deemed it desirable to compare different methods to prepare detached tree branches, and to select the best option for measuring photosynthesis of tall tree species *in vitro*.

We selected 10 common tree species as experimental materials. We tried to find a better* in vitro* method for more accurate and reliable field measurement of photosynthesis in tall trees through the comparison of *in vitro* photosynthesis measurements obtained from tree branches prepared by five different methods (branch beveling, branch cracking, branch splitting, branch girdling, and branch immersion in 2 mmol/L salicylic acid solution) with *in situ* photosynthesis measurements. The methods employed for the leaf photosynthesis measurements are shown in Table 1. These methods are meant to simulate in situ characteristics of well-watered plants, and would not be expected to simulate in situ characteristics of plants under water stress.

**Table 1:** Methods employed for the leaf photosynthesis measurements.

Treatment	Treatment description
In situ	Connect with mother plant (CK).
Beveling method	Current-year branches were beveled from the incision.
Cracking method	End (about 3 cm from the cut) of current-year branches was cracked.
Splitting method	End (about 3 cm from the cut) of current-year branches was split; then a small stone was inserted into the incision.
Girdling method	Phloem (about 3 cm from the cut) of current-year branches was girdled.
Salicylic acid method	End (about 3 cm from the cut) of current-year branches was immersed in 2 mmol/L salicylic acid (SA) solution.

Specifically, we aimed to address two main questions: (i) To what extent can photosynthetic parameters of trees, measured *in vitro*, substitute for *in situ* measurements of those same parameters? (ii) Which of the five methods used to prepare tree branches is more reliable and convenient for *in vitro* photosynthesis measurement in trees?

## Materials and Methods

### Experimental materials

All species under study were tree species grown at Xi’an University, in the southern suburb of Xi’an city, Shaanxi Province, China. Xi’an belongs to the warm temperate monsoon zone and it has a semi-humid continental climate. Annual average total precipitation, mean annual temperature and humidity are 613.8 mm, 13.3 °C, and 69.6%, respectively. Rainfall is typically, unevenly distributed, concentrating in July, August, September, and October. The average number of sunshine hours is about 1900 h per year, and the frostless period is about 220 d. Meteorological data were collected from the China Meteorological Administration Scientific Data Sharing Center (http://www.weather.com.cn/cityintro/101110101.shtml). The general information on the experimental tree species is shown in Table 2. All selected tree species are well-watered common species, including evergreen and deciduous tree species.

**Table 2:** The general information of the tested tree species. The data are means ± standard deviations (*n* = 3).

Trees	Abbrev	Height (m)	Diameter at breast height (cm)	Crown width (m)
*Cerasus yedoensis*	CY	5.2 ± 0.3	11.3 ± 1.1	3.2 ± 0.3
*Magnolia denudata*	MD	6.3 ± 0.4	12.5 ± 1.6	2.9 ± 0.2
*Hibiscus syriacus*	HS	3.6 ± 0.3	6.8 ± 0.9	1.9 ± 0.2
*Populus tomentosa*	PT	15.1 ± 0.8	38.9 ± 3.5	7.2 ± 0.4
*Acer elegantulum*	AE	7.7 ± 0.6	13.9 ± 1.8	4.4 ± 0.5
*Koelreuteria paniculata*	KP	10.6 ± 0.9	35.8 ± 3.1	9.1 ± 0.9
*Diospyros kaki*	DK	8.9 ± 0.7	16.8 ± 1.9	7.8 ± 0.7
*Aesculus chinensis*	AC	5.2 ± 0.5	7.6 ± 0.8	2.0 ± 0.1
*Eriobotrya japonica*	EJ	3.7 ± 0.2	9.8 ± 1.2	4.1 ± 0.3
*Ligustrum lucidum*	LL	7.9 ± 0.9	22.1 ± 2.7	5.4 ± 0.7

### Pretreatment before determination

The five methods used for preparing samples for *in vitro* photosynthesis measurement of the selected 10 tree species are shown in Table 2. Among these methods, beveling, cracking, splitting, and girdling are illustrated in Fig. S1, so as to provide a clear idea of what they involve.

Measurements were performed on sunny days in September of 2016. Healthy mature branches in the upper part of the sunny side were selected from at least three, similarly sized trees previously marked. Branches of the same tree species were selected for similarity in diameter and growth. They were all cut to a length of about 50 cm.

In order to avoid air getting into the xylem vessels at the incision point, which would hinder water absorption, cut branches were immersed in water immediately and the ends (about 4 cm) were cut again with a knife under the water. All selected species were broad-leaved trees, and the leaves measured could occupy the full leaf chambers. The leaf chamber area was 6 cm^2^.

### Photosynthetic response curves

Photosynthetic response curves were measured with a Li-6400 Portable Photosynthesis System (Li-Cor, Lincoln, NE, USA). Before measurement, each sample leaf was illuminated with a saturating level of PPFD for about 25 minutes to achieve fully photosynthetic induction. All measurements were conducted on fully-expanded, recently matured leaves at a CO_2_ concentration of 400 ppm. Li-Cor CO_2_ injector system was used for CO_2_ source. Relative humidity of the air in the leaf chamber was controlled at ≈70% and leaf temperature at 25 °C. Photosynthetically active radiation (PAR) was 1,800, 1,500, 1,200, 1,000, 800, 500, 200, 120, 100, 50, 30, 20, and 0 μmol m^−2^ s ^−1^. Steady-state values from each leaf were recorded after 200 s equilibration period under each PPFD level. Three branches of the same tree species were selected randomly for similarity in diameter and growth among three equal sized trees.

### Measurements were made in triplicate for each treatment

Maximum net photosynthetic rate (*P*_max_) was fitted by the least square method according to the following empirical equation (Bassman & Zwier, 1991):

\begin{eqnarray*}{P}_{\mathrm{n}}={P}_{\mathrm{max}}(1-{C}_{0}{e}^{(-\alpha \mathrm{PAR/ Pmax})}) \end{eqnarray*}

where, *α* is the apparent quantum efficiency under weak light, *C*_0_ is an index to measure the net photosynthetic rate approaching zero under weak light.

The light compensation point (LCP) and light saturation point (LSP) were calculated according to the following formulas:

LCP = *P*_max_ln(C_0_)/*α*

LSP = *P*_max_ln(100×C_0_)/*α*

In addition, dark respiration rate (*R*_d_) was estimated by the intercept of the light response curve with the axis of net photosynthetic rate.

### Gas exchange

After the light response curves were taken, we compared the above indexes for the different methods and chose the better ones for *in vitro* gas exchange measurements. Transpiration rate (Tr, mmol H_2_O m^−2^ s^−1^), stomatal conductance (g_−1s_, mmol H_2_O m^−2^ s^−1^) and intercellular CO_2_ concentration (Ci, μmol mmol^−1^) were directly calculated by using the LI-6400 portable photosynthesis system at a CO_2_ concentration of 400 ppm. The size of branches and leaf selection were the same as above. Tr, g_s_ as well as Ci of all tree species were measured under their respective saturated light intensity (Table  S1). Measurements for each parameter were taken three times. Furthermore, we compared Tr, g_s_, and Ci between the better branch detachment method for *in vitro* measurements and the *in situ* measurement to estimate the water status of the branches in each case.

### Statistical analysis

Normality of error and homogeneity of variance were checked. Differences among 5 branch detachment methods were analyzed with one-way ANOVA followed by Duncan’s multiple range test. All analyses and regressions were carried out using STATISTICA 6.0 (Statsoft, Tulsa, OK, USA). The graphing software used was Origin 8.0 (Origin lab, Northampton, USA).

## Results

As shown in Table 3, after immersion in SA solution, the *in vitro P*_max_ values of all 10 tree species were not significantly different from the *in situ P*_max_ values. After branches were cracked or girdled, *in vitro P*_max_ of eight and seven out of the 10 experimental species, respectively, were similar to *in situ P*_max_ values. However, when the customary beveling method was used, only four tree species had similar to in vitro and *in situ* values, whereas the *P*_max_ values of the other six tree species were significantly lower than the *P*_max_ values measured *in situ*. A similar trend was observed for splitting. The ratio of *in vitro P*_max_ to *in situ P*_max_ also showed that *P*_max_ of most tree species reached 90% to 110% by immersion in SA and the cracking methods.

**Table 3:** Maximum photosynthesis rate (*P*_*max*_) of 10 tree species under different treatments. The data are means ± standard errors (*n* = 3). Different lowercase letters in the same row indicate significant differences among different treatments at *P* < 0.05 level. The values in parentheses following the data are the percentage of *in-vitro P*_*max*_ to *in-situ P*_*max*_.

Tree species	In Situ		Beveling		Cracking		Splitting		Girdling		Immersing in SA	
	µmol (CO_2_) m^−2^ s^−1^		µmol (CO_2_) m^−2^ s^−1^		µmol (CO_2_) m^−2^ s^−1^		µmol (CO_2_) m^−2^ s^−1^		µmol (CO_2_) m^−2^ s^−1^		µmol (CO_2_) m^−2^ s^−1^	
*Cersus yedoensisa*	8.50 ± 0.22	a	6.26 ± 0.54 (74)	b	7.84 ± 0.24 (92)	ab	8.18 ± 0.21(96)	a	7.43 ± 0.26(87)	ab	8.72 ± 1.18(103)	a
*Magnolia denudata*	3.24 ± 0.02	a	2.95 ± 0.37 (91)	ab	3.25 ± 0.68(100)	a	2.84 ± 0.20(88)	b	4.14 ± 0.15(128)	a	3.56 ± 0.44(110)	a
*Hibiscus syriacus*	9.93 ± 1.69	a	8.97 ± 0.64 (90)	a	9.57 ± 0.07(96)	a	7.52 ± 0.36(76)	a	7.80 ± 0.19(79)	a	9.16 ± 0.42(92)	a
*Populus tomentosa*	13.70 ± 1.03	a	9.62 ± 1.34 (70)	b	15.16 ± 1.38(111)	a	10.25 ± 0.35(75)	b	10.29 ± 0.75(75)	b	12.39 ± 0.40(90)	ab
*Acer elegantulum*	8.66 ± 1.35	a	6.28 ± 0.84(73)	ab	7.35 ± 0.44(85)	a	6.04 ± 0.66 (70)	b	7.96 ± 0.81(92)	a	7.77 ± 0.63(90)	a
*Koelreuteria paniculata*	11.31 ± 1.39	a	4.00 ± 0.13 (35)	c	11.16 ± 1.17(99)	a	6.68 ± 0.18(59)	b	5.40 ± 0.55(48)	b	9.83 ± 0.29(87)	a
*Diospyros kaki*	14.05 ± 1.26	a	8.56 ± 0.93 (61)	b	9.01 ± 2.03(64)	b	8.59 ± 0.41(61)	b	13.43 ± 1.60(96)	a	11.00 ± 0.80(78)	ab
*Aesculus chinensis*	6.11 ± 0.27	ab	7.43 ± 0.56 (122)	a	5.40 ± 0.40(88)	ab	4.46 ± 0.77(73)	b	6.43 ± 0.74(105)	a	6.33 ± 0.35(104)	a
*Eriobotrya japonica*	13.53 ± 0.68	a	9.75 ± 1.48 (72)	b	7.83 ± 0.56(58)	c	9.64 ± 0.48(71)	b	12.04 ± 1.36(89)	a	13.92 ± 1.19(103)	a
*Ligustrum lucidum*	9.99 ± 1.02	a	7.29 ± 1.06 (73)	b	10.70 ± 0.91(107)	a	6.11 ± 0.10(61)	b	7.18 ± 0.64(72)	b	11.18 ± 0.73(112)	a

In branches beveled or cracked, *in vitro R*_d_ of eight and nine, out of the 10 tree-species under study, respectively, was similar to *in situ R*_d_ (Table 4). As for splitting and girdling, *in vitro R*_d_ of five and eight, out of the 10 tree-species tested, respectively, was similar to *in situ R*_d_ (Table 4). On the other hand, under SA treatment, only four tree-species maintained similar *R*_d_, whereas most of the remaining tree species (*Cerasus yedoensis*,* Magnolia denudata, Hibiscus syriacus*,* Diospyros kaki*, *Ligustrum lucidum*), showed significantly higher *R*_d_.

**Table 4:** Dark respiration rate (*R*_d_) of 10 kinds of trees under different treatments. The values are means ± standard errors (*n* = 3). Different lowercase letters in the same row indicate significant differences among different treatments at *P* < 0.05 level. The values in parentheses following the data are the percentage of *in-vitro R*_d_ to *in-situ R*_d_.

Tree species	In Situ µmol CO_2_ m^−2^ s^−1^		Beveling µmol CO_2_ m^−2^ s^−1^		Cracking μmol CO_2_ m^−2^ s^−1^		Splitting μmol CO_2_ m^−2^ s^−1^		Girdling μmol CO_2_ m^−2^ s^−1^		Immersing in SA μmol CO_2_ m^−2^ s^−1^	
*Cerasus yedoensis*	0.69 ± 0.10	c	0.95 ± 0.12(138)	bc	0.99 ± 0.07(143)	bc	1.08 ± 0.13(157)	b	0.68 ± 0.11(99)	c	1.60 ± 0.10(232)	a
*Magnolia denudata*	0.28 ± 0.06	bc	0.39 ± 0.10(139)	b	0.36 ± 0.07(129)	b	0.18 ± 0.04(64)	c	0.36 ± 0.08(129)	b	0.55 ± 0.18(196)	a
*Hibiscus syriacus*	0.59 ± 0.13	b	0.70 ± 0.17(118)	b	0.79 ± 0.08(134)	b	0.81 ± 0.21(137)	b	0.64 ± 0.15(108)	b	1.39 ± 0.39(236)	a
*Populus tomentosa*	0.89 ± 0.10	ab	0.63 ± 0.03(71)	ab	0.81 ± 0.21(91)	ab	1.05 ± 0.07(118)	a	0.40 ± 0.10(45)	b	0.82 ± 0.11(92)	ab
*Acer elegantulum*	0.73 ± 0.05	a	0.82 ± 0.20(112)	a	0.97 ± 0.21(133)	a	0.69 ± 0.14(95)	a	0.73 ± 0.13(100)	a	0.76 ± 0.20(230)	a
*Koelreuteria paniculata*	0.65 ± 0.18	b	1.09 ± 0.27(167)	a	0.58 ± 0.14(89)	b	1.05 ± 0.23(162)	a	0.75 ± 0.10(115)	b	0.73 ± 0.18(112)	b
*Diospyros kaki*	0.28 ± 0.06	c	0.61 ± 0.09(218)	a	0.28 ± 0.05(100)	c	0.46 ± 0.12(164)	b	0.45 ± 0.11(161)	b	0.55 ± 0.16(196)	ab
*Aesculus chinensis*	0.64 ± 0.08	a	0.49 ± 0.11(76)	ab	0.44 ± 0.05(68)	ab	0.34 ± 0.09(53)	b	0.45 ± 0.04(70)	ab	0.26 ± 0.04(41)	c
*Eriobotrya japonica*	0.92 ± 0.18	ab	0.60 ± 0.15(65)	b	0.64 ± 0.19(69)	b	0.75 ± 0.04(82)	b	0.66 ± 0.16(72)	b	1.19 ± 0.23(129)	a
*Ligustrum lucidum*	0.79 ± 0.21	b	1.12 ± 0.20(142)	b	1.88 ± 0.52(238)	a	0.53 ± 0.07(67)	c	0.49 ± 0.06(62)	c	2.05 ± 0.36(259)	a

None of the five methods had any significant effect on apparent quantum efficiency (AQE) of most tree species studied (Table 5). It is noteworthy that the SA treatment resulted in a significant decrease of *in vitro* AQE in *Cerasus yedoensis*, *Hibiscus syriacus* and* Eriobotrya japonica*.

**Table 5:** Apparent quantum efficiency (AQE) of 10 kinds of trees under different treatments. The values are means ± standard errors (*n* = 3). Different lowercase letters in the same row indicate significant differences among different treatments at *P* < 0.05 level. The values in parentheses following the data are the percentage of *in-vitro* AQE to *in-situ* AQE.

Tree species	In Situ mol mol^−1^		Beveling mol mol^−1^			Cracking mol mol^−1^		Splitting mol mol^−1^		Girdling mol mol^−1^		Immersing in SA mol mol^−1^		
*Cerasus yedoensis*	0.057 ± 0.004	ab		0.069 ± 0.004(121)	a	0.054 ± 0.005(95)	b	0.053 ± 0.001(93)	b	0.060 ± 0.002(105)	a		0.039 ± 0.006(68)	c
*Magnolia denudata*	0.044 ± 0.005	ab		0.048 ± 0.007(109)	ab	0.030 ± 0.005(68)	b	0.051 ± 0.008(116)	a	0.028 ± 0.004(63)	b		0.033 ± 0.009(75)	b
*Hibiscus syriacus*	0.075 ± 0.001	ab		0.083 ± 0.004(111)	a	0.066 ± 0.003(88)	b	0.070 ± 0.005(93)	b	0.077 ± 0.002(103)	ab		0.0480 ± 0.006(64)	c
*Populus tomentosa*	0.054 ± 0.005	a		0.062 ± 0.009(115)	a	0.061 ± 0.004(113)	a	0.051 ± 0.003(94)	a	0.065 ± 0.001(120)	a		0.053 ± 0.005(98)	a
*Acer elegantulum*	0.050 ± 0.004	a		0.044 ± 0.001(88)	a	0.051 ± 0.004(102)	a	0.046 ± 0.004(92)	a	0.047 ± 0.003(94)	a		0.041 ± 0.005(82)	a
*Koelreuteria paniculata*	0.049 ± 0.004	a		0.050 ± 0.007(102)	a	0.055 ± 0.003(112)	a	0.058 ± 0.004(118)	a	0.053 ± 0.001(108)	a		0.049 ± 0.000(100)	a
*Diospyros kaki*	0.044 ± 0.004	a		0.059 ± 0.003(134)	a	0.044 ± 0.006(100)	a	0.062 ± 0.007(141)	a	0.047 ± 0.003(107)	a		0.061 ± 0.007(139)	a
*Aesculus chinensis*	0.035 ± 0.003	ab		0.041 ± 0.004(117)	ab	0.027 ± 0.005(77)	b	0.045 ± 0.003(129)	ab	0.049 ± 0.004(140)	a		0.034 ± 0.002(97)	ab
*Eriobotrya japonica*	0.066 ± 0.002	a		0.053 ± 0.003(80)	b	0.053 ± 0.001(80)	b	0.056 ± 0.001(85)	b	0.059 ± 0.002(89)	b		0.058 ± 0.003(88)	b
*Ligustrum lucidum*	0.053 ± 0.006	a		0.055 ± 002(104)	a	0.058 ± 0.006(109)	a	0.044 ± 0.003(83)	a	0.058 ± 0.003(109)	a		0.049 ± 0.002(92)	a

Branch cracking had hardly any effect on light compensation point (LCP) (Table 6). As for beveling and immersion in SA, *in vitro* LCP of six, out of the 10 tree-species tested, was similar to *in situ* LCP. When cracking was used, except for* Magnolia denudata*, LSP of all tree species under study was similar to the LSP value measured *in situ* (Table 7).

**Table 6:** Light compensation point (LCP) of 10 kinds of trees under different treatments. The values are means ± standard errors (*n* = 3). Different lowercase letters in the same row indicate significant differences among different treatments at *P* < 0.05 level.

Tree species	In Situ μmol m^−2^ s^−1^		Beveling μmol m^−2^ s^−1^		Cracking μmol m^−2^ s^−1^		Splitting μmol m^−2^ s^−1^		Girdling μmol m^−2^ s^−1^		Immersing in SA μmol m^−2^ s^−1^	
*Cerasus yedoensis*	17.87 ± 2.02	b	12.44 ± 2.76	c	20.56 ± 0.69	b	19.20 ± 1.85	b	10.91 ± 2.22	c	28.73 ± 5.87	a
*Magnolia denudata*	8.60 ± 1.78	a	10.52 ± 1.07	a	11.59 ± 2.04	a	8.58 ± 2.05	a	11.05 ± 2.56	a	11.64 ± 3.14	a
*Hibiscus syriacus*	9.36 ± 1.78	bc	7.65 ± 1.59	c	11.48 ± 0.73	b	12.47 ± 2.65	b	7.79 ± 1.89	c	19.06 ± 3.53	a
*Populus tomentosa*	15.93 ± 0.95	a	10.28 ± 1.23	b	12.51 ± 3.15	ab	19.58 ± 0.23	a	6.50 ± 1.49	c	15.02 ± 2.32	a
*Acer elegantulum*	12.68 ± 1.44	a	15.35 ± 4.96	a	18.16 ± 4.51	a	12.59 ± 2.94	a	15.01 ± 3.49	a	17.04 ± 2.98	a
*Koelreuteria paniculata*	12.33 ± 3.11	a	15.17 ± 2.46	a	12.60 ± 3.84	a	16.65 ± 2.67	a	13.44 ± 1.98	a	14.38 ± 3.53	a
*Diospyros kaki*	6.48 ± 1.64	b	11.87 ± 2.23	a	6.53 ± 2.13	b	7.15 ± 1.37	b	6.78 ± 0.76	b	6.97 ± 1.67	b
*Aesculus chinensis*	11.68 ± 4.08	a	12.07 ± 3.23	a	10.34 ± 2.60	a	7.59 ± 2.05	b	8.93 ± 0.36	ab	7.20 ± 2.59	b
*Eriobotrya japonica*	13.61 ± 2.95	a	10.45 ± 2.77	a	11.71 ± 2.67	a	12.86 ± 0.48	a	10.86 ± 2.32	a	15.01 ± 3.06	a
*Ligustrum lucidum*	14.97 ± 4.20	b	18.68 ± 2.58	a	22.23 ± 6.54	a	11.52 ± 1.03	bc	9.81 ± 1.16	c	23.10 ± 5.20	a

**Table 7:** Light saturation point (LSP) of 10 kinds of trees under different treatments. The values are means ± standard errors (*n* = 3). Different lowercase letters in the same row indicate significant differences among different treatments at *P* < 0.05 level.

Tree species	In Situ *μ*mol m^−2^ s^−1^		Beveling *μ*mol m^−2^ s^−1^		Cracking *μ*mol m^−2^ s^−1^		Splitting *μ*mol m^−2^ s^−1^		Girdling *μ*mol m^−2^ s^−1^		Immersing in SA *μ*mol m^−2^ s^−1^	
*Cerasus yedoensis*	711.32 ± 57.26	b	432.45 ± 38.49	c	703.64 ± 82.70	b	735.73 ± 29.87	b	582.94 ± 41.79	bc	1,067.50 ± 49.02	a
*Magnolia denudata*	358.63 ± 45.89	bc	310.90 ± 74.84	c	514.86 ± 33.38	ab	281.60 ± 67.68	c	438.80 ± 114.59	b	609.48 ± 160.12	a
*Hibiscus syriacus*	614.48 ± 91.43	b	508.07 ± 53.87	b	679.96 ± 29.18	b	515.19 ± 51.96	b	476.09 ± 10.26	b	943.48 ± 116.67	a
*Populus tomentosa*	1,191.80 ± 35.32	a	730.07 ± 16.73	b	1,151.50 ± 106.26	a	954.23 ± 87.80	ab	732.93 ± 55.35	b	1,107.60 ± 130.12	a
*Acer elegantulum*	840.69 ± 94.89	a	648.65 ± 78.72	a	694.25 ± 86.90	a	659.19 ± 139.07	a	784.19 ± 32.29	a	890.70 ± 40.57	a
*Koelreuteria paniculata*	908.50 ± 159.10	a	962.58 ± 45.71	a	844.31 ± 48.29	a	854.59 ± 19.18	a	884.68 ± 42.13	a	941.74 ± 25.96	a
*Diospyros kaki*	886.50 ± 121.41	b	1,197.70 ± 110.77	a	969.48 ± 196.94	b	960.99 ± 64.09	b	980.40 ± 152.86	b	920.19 ± 10.60	b
*Aesculus chinensis*	859.74 ± 60.81	a	759.50 ± 115.55	a	706.60 ± 202.73	a	475.05 ± 108.21	b	629.53 ± 96.20	ab	436.01 ± 94.90	b
*Eriobotrya japonica*	958.54 ± 21.97	ab	863.65 ± 128.23	ab	696.89 ± 60.06	b	963.79 ± 118.62	ab	803.26 ± 42.94	b	1,129.90 ± 94.51	a
*Ligustrum lucidum*	636.76 ± 111.08	bc	898.16 ± 88.61	b	879.53 ± 89.21	b	655.93 ± 38.44	bc	574.33 ± 26.18	c	1,104.60 ± 98.04	a

**Figure 1:**
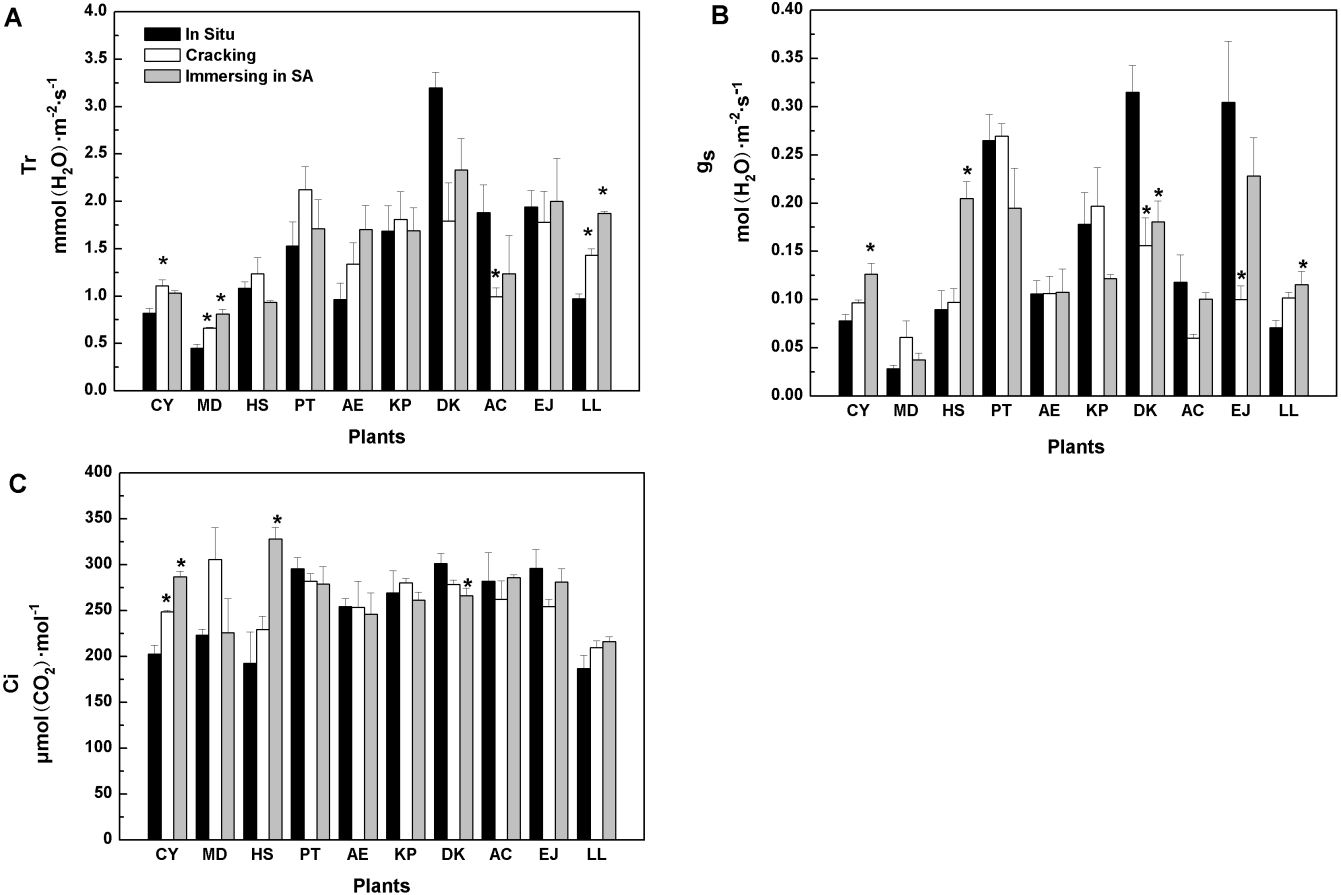
Effects of three treatments on (A) transpiration rate (Tr), (B) stomatal conductance (g…), (C) intercellular CO_2_ concentration (Ci) of 10 kinds of trees. The values are means (*n* = 3). Error bars show standard errors. Asterisks denote significant differences (*P* < 0.05) between the *in-vitro* methods and *in-situ* method. The abbreviations of 10 kinds of trees are shown in Table 2.

Similarly, when cracking was used, six tree species showed similar *in vitro* Tr values to *in situ* values, while three species (*Cerasus yedoensis*,* Magnolia denudata*, and *Ligustrum lucidum*), registered higher Tr, whereas *Diospyros kaki* showed lower Tr values, compared with *in situ* measurements (Fig. 1A). SA treatment caused higher Tr in *Magnolia denudate* and *Ligustrum lucidum*, while it had no effects on the rest of the species.

As shown in Fig. 1B, *g*_s_ values of eight tree-species were not significantly influenced by cracking, while six were not affected by SA treatment. Furthermore, when cracking and immersion in SA were used, *in vitro* C_i_ of nine and seven, out of the 10 tree-species, respectively, was similar to C_i_ measured *in situ* (Fig. 1C).

## Discussion

As crucial components of global leaf economic spectrum, photosynthetic parameters, especially maximum net photosynthetic rate (*P*_max_) is the most important physiological trait of the leaf (Wright et al., 2004; Marino, Aqil & Shipley, 2010). In this study, we focused on developing a reliable and convenient method to measure photosynthetic parameters for tall trees, which is difficult to conduct *in situ*, on the intact tree. As intact foliage measurement is difficult, an alternative protocol consists of cutting branches with leaves and then cutting once more underwater (i.e., what we call beveling method here), which has become a standardized protocol (Perez-Harguindeguy et al., 2013). However, when branches are cut off, water supply to the leaves is vulnerable to xylem cavitation and embolism, which may result in underestimated values of *P*_max_ (Edwards & Jarvis, 1982; Gullo & Salleo, 1992; Luo, Zhang & Zhang, 2016). Photosynthesis may be severely hindered by water deficit (Ghotbi-Ravandi et al., 2014; Johnson, 2016) due to stomata closure (Saliendra, Sperry & Comstock, 1995; Boyle, Mcainsh & Dodd, 2015) and to decrease of Rubisco activity (Flexas & Medrano, 2002). Therefore, preserving water supply is the top priority, while attempting *in vitro* measurement of photosynthesis in cut branches from tall trees. As maximum net photosynthetic rate (*P*_max_) and dark respiration rate (*R*_d_) are the two opposite extremes of the static photosynthetic light–response curve, they are the most important criteria in the evaluation of *in vitro* methods for measuring photosynthesis. We found that *P*_max_ of 6 tree species was significantly lower than the corresponding *in situ* values when branch beveling preceded measurements (Table 3). Since *P*_max_ directly reflects plant photosynthetic potential (Sikder et al., 2015), it most adequately reveals the limitations of the beveling method to match *in situ* results of photosynthetic potential of the tree species under study. Further, after using the beveling method, four tree species displayed significant differences in LCP with respect to *in situ* values. It shows that beveling is prone to error in assessing photosynthetic characteristics of some trees.

Among the five methods evaluated, cracking proved better than any other. When two tree species (*Diospyros kaki, Eriobotrya japonica*) were removed, the results of regression *in situ P*_max_ against *in vitro P*_max_ were better (*R*^2^ =0.96) (Table S2)**.**Regarding *P*_max_, *R*_d_, and AQE, cracking was a good substitute for *in situ* measurement of photosynthetic parameters (Tables 3,  4, and 5). The water status of detached tree branches is an important premise for measuring photosynthesis *in vitro*. As long as a cut branch can obtain water promptly, cavitation and embolism can be effectively prevented (Tyree & Dixon, 1986). Therefore, we further studied the water status of branches after cracking. There was no significant difference between *in vitro* (i.e., with cracking method) and *in situ* transpiration rates (Tr) for most tree species studied (Fig. 1A). Transpiration rate is one of the most common physiological indexes used to measure the magnitude of water deficit experienced by leaf tissues (Thomas et al., 2010; Belko et al., 2013). Variations of Tr indicate the environmental adaptability of plants (Monteiro et al., 2016). Generally, Tr will be higher when plant water status is non-limiting. Hence, our results showed that branches of most trees studied could effectively continue to get a continuous water supply by using the cracking method for detachment from the tree prior to gas exchange and photosynthesis measurements. Stomatal conductance (g_s_) is another indicator of water, energy and CO_2_ cycles between plants and the atmosphere (Roche, 2015; Saradadevi et al., 2016). Plants can vary the size of stomata to adapt to the environment. It is well known that intercellular CO_2_ concentration (Ci) is determined by stomatal and non-stomatal factors (Farquhar & Sharkey, 1982). When the concentration of CO_2_ in the air remains constant, Ci is determined by stomatal conductance, mesophyll conductance and photosynthetic activity of mesophyll cells (Farquhar & Sharkey, 1982). For most of our 10 species, *in vitro* measurements following branch cracking did not change g_s_ nor Ci, compared with* in situ* determinations (Figs. 1B, 1C), suggesting that cracking pre-treatment could ensure effective, continued water supply to detached branches; thus, maintaining photosynthetic activity. In contrast, as far as *P*_max_ is concerned, the splitting method for branch detachment did not work very well. Similarly, overall, the results of the girdling method were inferior to those of the cracking method.

Salicylic acid, a signaling molecule, has direct or indirect effects on many physiological processes in plants by its interaction with some functional or other signaling molecules (Khan, Prithiviraj & Smith, 2003; Sánchez-Rojo et al., 2015). Regarding *P*_max_, immersion of the branch in SA solution may also reproduce *in situ* measurements. Water status and *P*_max_ of branches immersed in SA solution were close to the corresponding *in situ* values. (Table 3, Fig. 1). However, after immersing twigs in salicylic acid, *R*_d_ rates of some trees became larger than the corresponding *in situ* values (Table 4). Accurate estimation of *R*_d_ is related to the calculation of some important photosynthetic parameters, such as photorespiration rate and electron flux distribution (Burris, 1977; Mebrahtu et al., 2011); thus, the SA immersion method likely had some negative effects in the determination of *in vitro* photosynthesis, since SA can not only regulate stomatal opening, RubisCO, PEPC and CA (carbonic anhydrase) activities, but also, it can regulate electron transport and photosynthetic energy conversion (Vicente & Plasencia, 2011). Several studies have shown that SA improves environmental adaptability of plants (e.g. Nazar et al., 2011; Janda et al., 2014). Under conditions of environmental stress, SA can inhibit peroxidation of unsaturated fatty acid (Moradkhani et al., 2012), restrain the decrease of photosynthetic pigment content (Fariduddin, Hayat & Ahmad, 2003) and ultimately, sustain higher photosynthetic rates to ensure the continuation of plant growth (Hayat, Fariduddin & Ali, 2005). However, the effects of salicylic acid on plant photosynthesis vary with environment, level of exogenous SA and plant species (Hayat, Fariduddin & Ali, 2005). These also explain why some traits of branches immersed in SA solution performed well, while some indexes performed poorly.

Among the five in-vitro methods, the SA method is linked with chemical regulation, while the other four ways (beveling method, cracking method, splitting method, girdling method) are associated with physical regulation. The physical methods in this experiment are easier to apply in field measurement than the chemical method because the latter necessitates preparation, preservation, and transportation of chemical reagents. Among the four physical methods, the cracking method is the best (Table 3, Fig. 1). In particular, when using the cracking method to measure photosynthetic characteristics of plants in the field, it is additionally necessary to carry branch scissors, a pair of pliers, about 2 L of pure water, and a small container, such as a small bucket. These tools and containers are light and portable, and therefore, the cracking method can be easily applied in the field.

Koike and Sakagami found that branch beveling could increase the absorption area of branches, and therefore, the method could be used to measure *in vitro* photosynthesis in most deciduous broad-leaved trees in Hokkaido (Koike & Sakagami, 1984; Koike, 1986). Tang & Wang (2011) found girdling was better than beveling in 7 major tree species of the temperate forest of northeastern China. However, the results of our study indicate that cracking was better with the 10 tree-species we used. We found that cracking effectively prevented water deficit (Fig. 1) and the *in vitro* photosynthetic activity observed in leaves was similar to that shown by leaves on the intact tree (Table 3).

Truly, the key to a reliable determination of *in vitro* photosynthesis is an uninterrupted, adequate, prompt water supply. The size of absorptive area directly restricts water absorption ability of detached branches. When using the cracking method, some phloem vessels at the end of the branch will be crushed as the branch is detached from the tree. Therefore, the result of cracking is equivalent to the superposition of removing terminal phloem and splitting the end more times. Evidently the water absorption area of cracked branches was the largest among the five methods under evaluation and, ultimately, water supply became more effective in this case.

In addition, in order to prevent xylem cavitation, all methods included a second cutting, which was carried out under water. As a result, for those species whose epidermal cells or phloem cells could secrete colloid fluids, the external force of cracking branches would increase the scouring action of water on colloid fluid remarkably. This likely had a positive effect as it may have prevented the blocking of the incision area. Altogether, these may be the reasons for the superior results obtained by the cracking method.

Compared to the *in situ* method, the cracking method yielded significantly underestimated *P*_max_ for *Diospyros kaki* and *Eriobotrya japonica* (Table 3), overestimated *R*_d_ for *Ligustrum lucidum* (Table 4), underestimated AQE for *Eriobotrya japonica* (Table 5), and overestimated LCP for *Ligustrum lucidum* (Table 6). Moreover, estimates of leaf transpiration under cracking method significantly differed from those obtained with the *in situ* method for *Cerasus yedoensis*, *Magnolia denudata*, *Aesculus chinensis*, and *Ligustrum lucidum* (Fig. 1). Finally, estimates of stomatal conductance under the cracking method significantly differed from those obtained with the *in situ* method for *D. kaki* and *E. japonica* (Fig. 1). This means that some tree species (*D. kaki, E. japonica, and L. lucidum*) appeared to be particularly sensitive to the cracking method, with differences in the performance of different trees for the same method. This phenomenon may be due to the differences in stomatal opening speed, anatomical structure of wood, and tree species characteristics (ring or diffuse porous wood) (Fay & Knapp, 1995; McCulloh et al., 2010; Jacobsen et al., 2012). It is necessary to further study on how these characteristics affect the test results in the future.

Although the 10 tree-species selected in this study are common species which include deciduous and evergreen species, it should be noted that water absorption capacity and water holding capacity of *in vitro* branches were not the same among tree species treated with the same method (Jacobsen et al., 2012). Therefore, further work is needed to examine more tree species.

## Conclusions

Photosynthesis is the determining factor of primary productivity and ecosystem function. In the case of tall trees in the field, branch beveling is a popular method used for the preparation of *in vitro* photosynthesis measurement of detached branches. However, some studies, as well as our own study, have found that this method is likely to underestimate the photosynthetic potential of some trees.

The key to the reliable and reproducible determination of *in vitro* photosynthesis is the prompt, adequate, and uninterrupted, supply of water to detached branches. The size of the absorption area directly restricts the water absorption ability of detached branches. Water absorption area of cracked branches was the largest among the five methods tested and ultimately, water supply was best achieved by cracking. Among the five methods tested for preparing the *in vitro* measurement of photosynthesis in tall trees, cracking proved bestfollowed by SA solution. It needs to be pointed out that there are differences in the performance of different trees for the same method and there were species (*D. kaki* and *E. japonica*) in which “cracking” did not yield an estimate close to the one obtained *in situ*.

Our findings provide practical methodological support for comprehensive, reliable, and accurate measurement of plant functional traits. Using the cracking—method to detach tree branches allows for measuring photosynthetic parameters *in vitro* more accurately, which in turn can yield more reliable data to use for the analysis of trade-off strategies at the leaf level.

##  Supplemental Information

10.7717/peerj.5933/supp-1 Data S1 Raw data of Fig. 1

10.7717/peerj.5933/supp-2 Figure S1 4 kinds of *in-vitro* ways employed for the leaf photosynthesis measurements (A) Beveling method: current-year branches were beveled from the incision. (B) Cracking method: end (about 3 cm from the cut) of current-year branches was cracked. (C) Splitting method: end (about 3cm from the cut) of current-year branches was split; then a small stone was inserted into the incision. (D) Girdling method: phloem (about 3 cm from the cut) of current-year branches was girdled. Salicylic acid method is easy to understand so that it doesn’t appear in Fig. S1.

10.7717/peerj.5933/supp-3 Table S1 The light saturation level of the tested tree species Tr, g_*s*_ as well as Ci of all tree species were measured under their respective saturated light intensity.

10.7717/peerj.5933/supp-4 Table S2 Regression *in situP*_*max*_ against *in vitroP*_*max*_ Cracking (*n* = 10) means the analysis results of all tested tree species. Cracking (*n* = 8) means the analysis results after removal of 2 tree species (*Diospyros kaki, Eriobotrya japonica*) whose ***in vitro*** *P*_*max*_ was significantly different with *in situ P*_*max*_.

## Additional Information and Declarations

### Data Availability

The following information was supplied regarding data availability: The raw data are provided in the Supplemental Files.
