# Supplementary figures and images for: Another choice for measuring tree photosynthesis in vitro
Source: PeerJ. 2019 Jan 8;7:e5933. doi: 10.7717/peerj.5933 (PMC6329340; doi:10.7717/peerj.5933)

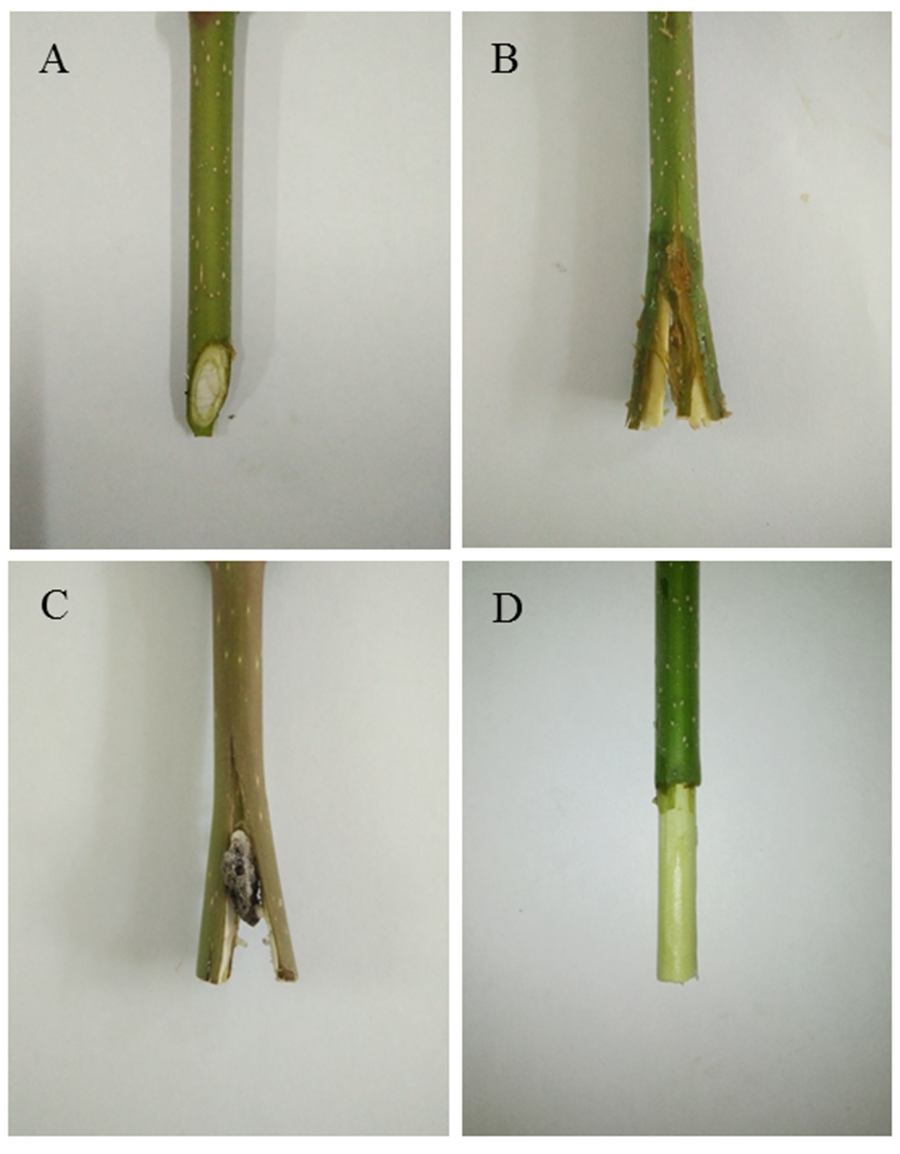

Supplement: Figure S1 — (A) Beveling method: current-year branches were beveled from the incision. (B) Cracking method: end (about 3 cm from the cut) of current-year branches was cracked. (C) Splitting method: end (about 3cm from the cut) of current-year branches was split; then a small stone was inserted into the incision. (D) Girdling method: phloem (about 3 cm from the cut) of current-year branches was girdled. Salicylic acid method is easy to understand so that it doesn’t appear in Fig. S1. [file peerj-07-5933-s002.png]
