# Supplementary material for: Another choice for measuring tree photosynthesis in vitro
Source: PeerJ. 2019 Jan 8;7:e5933. doi: 10.7717/peerj.5933 (PMC6329340; doi:10.7717/peerj.5933)
Supplement: Table S1 — Tr, gs as well as Ci of all tree species were measured under their respective saturated light intensity. [file peerj-07-5933-s003.doc]

| Trees | Abbrev | Light saturation point（μmol·m^-2^·s^-1^） |
| --- | --- | --- |
| *Cerasus yedoensis* | CY | 710 |
| *Magnolia denudata* | MD | 360 |
| *Hibiscus syriacus* | HS | 610 |
| *Populus tomentosa* | PT | 1200 |
| *Acer elegantulum* | AE | 840 |
| *Koelreuteria paniculata* | KP | 910 |
| *Diospyros kaki* | DK | 890 |
| *Aesculus chinensis* | AC | 860 |
| *Eriobotrya japonica* | EJ | 960 |
| *Ligustrum lucidum* | LL | 640 |
