# Supplementary material for: Another choice for measuring tree photosynthesis in vitro
Source: PeerJ. 2019 Jan 8;7:e5933. doi: 10.7717/peerj.5933 (PMC6329340; doi:10.7717/peerj.5933)
Supplement: Table S2 — Cracking (n = 10) means the analysis results of all tested tree species. Cracking (n = 8) means the analysis results after removal of 2 tree species (Diospyros kaki, Eriobotrya japonica) whose in vitro Pmax was significantly different with in situ Pmax. [file peerj-07-5933-s004.doc]

|  | In Situ  （n=10） | Beveling  （n=10） | Cracking  （n=10） | **Cracking**  **（n=8）** | Splitting  （n=10） | Girdling  （n=10） | Immersing in SA  （n=10） |
| --- | --- | --- | --- | --- | --- | --- | --- |
| R^2^ | 1 | 0.48 | 0.58 | **0.96** | 0.82 | 0.67 | 0.88 |
| *P* | 0.000 | 0.027 | 0.011 | **<0.001** | <0.001 | 0.004 | <0.001 |
